# Comorbidities prior to out-of-hospital cardiac arrest and diagnoses at discharge among survivors

**DOI:** 10.1136/openhrt-2023-002308

**Published:** 2023-11-14

**Authors:** Nellie Hjärtstam, Araz Rawshani, Gustaf Hellsén, Truls Råmunddal

**Affiliations:** 1 Department of Molecular and Clinical Medicine, University of Gothenburg, Goteborg, Sweden; 2 Cardiology, Sahlgrenska University Hospital, Goteborg, Sweden

**Keywords:** Coronary Artery Disease, Myocardial Infarction, Heart Failure, Out-of-Hospital Cardiac Arrest, Risk Factors

## Abstract

**Background:**

Out-of-hospital cardiac arrest (OHCA) has a dismal prognosis with overall survival around 10%. Previous studies have shown conflicting results regarding the prevalence and significance of comorbidities in OHCA, as well as the underlying causes. Previously, 80% of sudden cardiac arrest have been attributed to coronary artery disease. We studied comorbidities and discharge diagnoses in OHCA in all of Sweden.

**Methods:**

We used the Swedish Registry of Cardiopulmonary Resuscitation, merged with the Inpatient Registry and Outpatient Registry to identify patients with OHCA from 2010 to 2020 and to collect all their comorbidities as well as discharge diagnoses (among those admitted to hospital). Patient characteristics were described using means, medians and SD. Survival curves were performed among hospitalised patients with acute myocardial infarction (AMI) as well as heart failure.

**Results:**

A total of 54 484 patients with OHCA were included, of whom 35 894 (66%) were men. The most common comorbidities prior to OHCA were hypertension (43.6%), heart failure (23.6%), chronic ischaemic heart disease (23.6%) and atrial fibrillation (22.0%). Previous AMI was prevalent in 14.8% of men and 10.9% of women. Among women, 18.0% had type 2 diabetes, compared with 19.6% of the men. Among hospitalised patients, 30% were diagnosed with AMI, 27% with hypertension, 20% with ischaemic heart disease and 18% with heart failure as discharge diagnoses.

**Conclusion:**

In summary, we find evidence that nowadays a minority of cardiac arrests are due to coronary artery disease and AMIs and its complications. Only 30% of all cases of OHCA admitted to hospital were diagnosed with AMI. Coronary artery disease is now likely in the minority with regard to causes of OHCA.

**WHAT IS ALREADY KNOWN ON THIS TOPIC**

- Previous studies have shown conflicting results regarding the prevalence and significance of comorbidities in out-of-hospital cardiac arrest (OHCA), as well as the underlying causes. Previously, 50%–80% of sudden cardiac arrest have been attributed to coronary artery disease.

**WHAT THIS STUDY ADDS**

- We find evidence that nowadays a minority of cardiac arrests are due to coronary artery disease and its complications.

**HOW THIS STUDY MIGHT AFFECT RESEARCH, PRACTICE OR POLICY**

- Identify patients at risk for OHCA and prevent outcome with suitable medicines and lifestyle changes.

## Introduction

Out-of-hospital cardiac arrest (OHCA) affects 6000 people nationwide each year.^1^ The overall survival to hospital discharge after OHCA is around 10%.^2^ It is well known that survival in cardiac arrest is dependent on effective cardiopulmonary resuscitation (CPR) and prompt arrival of emergency medical services (EMS).^3–5^ However, comorbid diseases seem to be important for short-term and long-term mortality.^6^ Comorbidity is an increasing challenge in today’s healthcare, yet it is sparsely studied in OHCA, largely owing to difficulties recording comorbidities across the wide range of diagnoses and over longer periods of time.^6^

Previous studies show various results regarding the importance of comorbidity on survival. Due to heterogeneity between studies, comparisons are difficult but most studies show that comorbidity is associated with unfavourable outcome.^7 8^ However, some conditions, for example, myocardial infarction, have shown to be associated with lower mortality.^9^ Furthermore, there are no nationwide studies on the discharge diagnoses among individuals who are admitted after OHCA. It has traditionally been stated that 80% of sudden cardiac arrests are due to coronary artery disease.^10 11^ Whether this figure remains valid today is unknown, although recent studies demonstrated significantly lower rates.^12–14^ However, these studies have focused on specific subgroups and there is scarce data regarding the overall prevalence of acute coronary events and coronary artery disease in cases of OHCA. Given the reductions in coronary artery disease mortality in recent decades, it is plausible that a much lower proportion of OHCAs are due to coronary artery disease.

The aim of this study was to investigate the prevalence of individual comorbidities in OHCA, discharge diagnoses among survivors and the association between these diagnoses and survival.

## Methods

### Study population

We analysed all cases of OHCA retrospectively from 2010 to 2020 in the Swedish Registry for Cardiopulmonary Resuscitation (SRCR), a nationwide quality registry from 1990. The SRCR and its variables have been described in previous articles.^3^ The register complies with the Utstein style of reporting and OHCA cases are reported by the EMS. We included all cases of OHCA during the study period from 1 January 2010 to 31 December 2020 where resuscitation was initiated. OHCA is defined as cardiac arrest outside of the hospital walls. Only patients over 18 years of age were included in the study.

### Data linkage

To assess comorbidity, the SRCR data was merged with the Inpatient and Outpatient Registries. These registers are governmental nationwide databases that include the primary and secondary discharge diagnoses throughout Sweden. The Outpatient Registry contains data on all diagnoses established in outpatient clinics throughout Sweden since year 2002. The Inpatient Registry has been validated and contains all discharge diagnoses from inpatient records since 1987. The diagnoses in the registry are coded according to the Swedish International Classification of Disease (ICD).^15^ We retrieved all diagnoses in the patient registries up to 10 years prior to the cardiac arrest. Diagnoses established earlier than 10 years prior to the OHCA event were not available. Comorbidities were assessed as existing if one occurrence was noted in the registries.

The Swedish Prescribed Drug Registry was used to collect information on all drug prescriptions according to the Anatomical Therapeutic Chemical (ATC). We considered one prescription as intended use of a drug. We are aware that pharmacoepidemiological studies often use more granular definitions (eg, defined daily dose, rate and number of prescriptions, etc), but we opted for a simple method in this study.

Socioeconomic data from 1 year prior to the OHCA was collected from longitudinal integrated database for health insurance and labour market studies (LISA) database. All these databases, with exceptions of LISA, are held by the National Board of Health and Welfare. LISA is held by Statistics Sweden.

All of the above databases were merged with the SRCR using the unique 12-digit personal identity number administrated to all Swedish citizens.

### Discharge diagnosis

Patients who were hospitalised for OHCA also received a discharge diagnosis for the hospital admission. Therefore, patients who die in the prehospital setting or in the emergency room are not hospitalised and do not obtain a discharge diagnosis. We considered these discharge diagnosis as a proxy for the cause of cardiac arrest. The ICD code for cardiac arrest (I46) was ignored.

### Primary outcome

The primary outcome was 30-day survival.

### Statistical analysis

Baseline characteristics are described using means, medians and SD. Standardised mean difference (SMD) was used to compare men and women.^16^ SMD values of at least 0.10 were considered clinically relevant. Hypothesis tests were not performed on the baseline data, since it covers the entire population with OHCA in Sweden during the time period. Among those hospitalised, we also assessed the survival for patients with acute myocardial infarction (AMI; I21), as well as cases with heart failure (I25). Kaplan-Meier was used to generate survival curves.

## Results

### Baseline characteristics prior to cardiac arrest

Baseline data is provided in table 1. A total of 54 484 patients with OHCA were included in the study. The majority were men (n=35 894, 66%) with a mean age of 68 years. Women included in the study were older at baseline (72 years old). Most common drugs, used prior to OHCA, were anticoagulant or antiplatelet agent (ATC B01), beta blockers (ATC C07), ACE inhibitors (ATC C02) and diuretics (ATC C03). Lipid-lowering drugs were more common in men (25.5%) than women (19.9%). For further details, refer to online supplemental file 1.

10.1136/openhrt-2023-002308.supp1 Supplementary data

**Table 1:** Baseline characteristics in 54 484 patients with out-of-hospital cardiac arrest

Var	Men	Women	P value	SMD
n	35 894	18 590		
Patient characteristics				
Age—mean (SD)	67.98 (17.28)	71.53 (17.73)	<0.001	0.202
Cause of cardiac arrest (%)			<0.001	0.262
Heart disease	21 070 (65.8)	9313 (56.6)		
Overdose or intoxication	968 (3.0)	419 (2.5)		
Trauma or accident	862 (2.7)	262 (1.6)		
Pulmonary disease	1377 (4.3)	1341 (8.2)		
Suffocation	723 (2.3)	553 (3.4)		
Suicide	760 (2.4)	343 (2.1)		
Drowning	322 (1.0)	134 (0.8)		
Other	5929 (18.5)	4081 (24.8)		
Medications prescribed				
Anticoagulant or antiplatelet agent	13 467 (37.5)	6303 (33.9)	<0.001	0.075
Beta-blockers	11 773 (32.8)	6513 (35.0)	<0.001	0.047
ACE inhibitor or ARB	12 249 (34.1)	5616 (30.2)	<0.001	0.084
Diuretics	9080 (25.3)	5650 (30.4)	<0.001	0.114
Lipid lowering drugs	9266 (25.8)	3677 (19.8)	<0.001	0.144
Calcium channel blockers	5549 (15.5)	3154 (17.0)	<0.001	0.041
Antidiabetic drugs	5618 (15.7)	2525 (13.6)	<0.001	0.059
Initial presentation				
Initial rhythm (%)			<0.001	0.340
VF pVT	8841 (27.8)	2316 (14.2)		
PEA	5151 (16.2)	3127 (19.2)		
Asystole	17 772 (56.0)	10 882 (66.7)		
In-hospital interventions				
Coronary angiography performed	224 (43.8)	60 (24.6)	<0.001	0.412
PCI performed	2597 (34.6)	628 (18.1)	<0.001	0.382
CABG performed	161 (2.2)	23 (0.7)	<0.001	0.127
ICD implanted	973 (13.2)	216 (6.3)	<0.001	0.234
ECMO used	16 (3.1)	3 (1.3)	0.214	0.127

ARB, angiotensin receptor blockers; CABG, coronary artery bypass graft; ECMO, extracorporeal membrane oxygenation; ICD, implantable cardioverter defibrillator; PCI, percutaneous intervention; PEA, pulseless electrical activity; pVT, pulseless ventricular tachycardia; SMD, standardised mean difference; VF, ventricular fibrillation.

### Previous conditions

**Figure 1:**
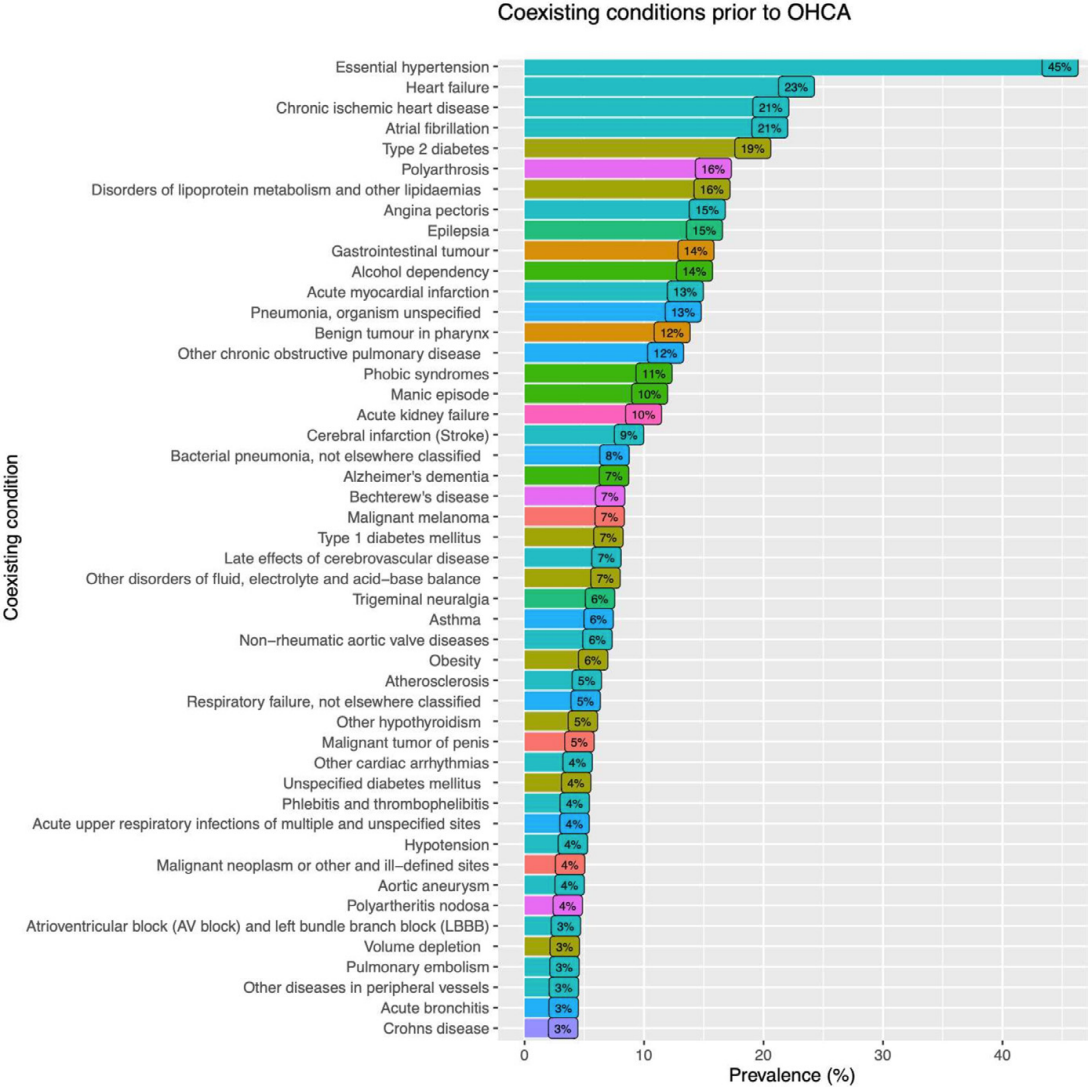
Prevalence of coexisting conditions prior to out-of-hospital cardiac arrest (OHCA) in a total of 54 484 patients (women and men).

The most common conditions prior to cardiac arrest among men were hypertension (43.6%), heart failure (23.6%), chronic ischaemic heart disease (23.6%) and atrial fibrillation (22.0%). Among women, 47.2% had hypertension, 21.1% heart failure and 17.6% atrial fibrillation. A history of AMI was prevalent in 14.8% of men and 10.9% of women. Among women, 18.0% had type 2 diabetes, compared with 19.6% of the men. Chronic obstructive pulmonary disease (COPD) was more common in women compared with men (14.6% vs 10.4%). Renal failure was more common in men (10.6% vs 8.8%). The prevalence of stroke was roughly equal in men and women (8.7% vs 8.8%, respectively). For further details, refer to table 2. Overall rates of comorbidities are presented in figure 1.

**Table 2:** Previous conditions in 54 484 patients with out-of-hospital cardiac arrest

Var	Men	Women	P value	SMD
n	35 894	18 590		
Previous conditions (%)				
Hypertension	15 637 (43.6)	8782 (47.2)	<0.001	0.074
Heart failure	8470 (23.6)	3927 (21.1)	<0.001	0.059
Chronic ischaemic heart disease	8462 (23.6)	2765 (14.9)	<0.001	0.222
Atrial fibrillation	7899 (22.0)	3277 (17.6)	<0.001	0.110
Type 2 diabetes	7053 (19.6)	3353 (18.0)	<0.001	0.041
Dyslipidaemia	6154 (17.1)	2393 (12.9)	<0.001	0.120
Angina including unstable angina	6100 (17.0)	2229 (12.0)	<0.001	0.143
Acute myocardial infarction	5299 (14.8)	2034 (10.9)	<0.001	0.114
Chronic obstructive pulmonary disease	3721 (10.4)	2711 (14.6)	<0.001	0.128
Renal failure	3790 (10.6)	1628 (8.8)	<0.001	0.061
Thrombotic stroke	3120 (8.7)	1642 (8.8)	0.593	0.005
Alzheimer’s dementia	2199 (6.1)	1885 (10.1)	<0.001	0.147
Type 1 diabetes mellitus	2599 (7.2)	1222 (6.6)	0.004	0.026
Subarachnoid haemorrhage	2483 (6.9)	1251 (6.7)	0.420	0.007

SMD, standardised mean difference.

### Outcomes

Overall, 12.5% of the men and 8.1% of the women were alive at 30 days following a cardiac arrest. For more details, refer to table 3.

**Table 3:** Outcomes in 54 568 patients with out-of-hospital cardiac arrest

Var	Men	Women	P value	SMD
Outcomes				
ROSC any ROSC	11 729 (34.2)	6173 (35.1)	0.057	0.018
CPC score at discharge			0.017	0.117
CPC score 1 no sequelae	2576 (76.7)	792 (72.9)		
CPC score 2 mild sequelae	490 (14.6)	183 (16.8)		
CPC score 3 moderate sequelae	213 (6.3)	73 (6.7)		
CPC score 4 severe sequelae	67 (2.0)	37 (3.4)		
CPC score 5 brain death	11 (0.3)	2 (0.2)		
Survival at 30 days	4495 (12.5)	1512 (8.1)	<0.001	0.145

CPC, cerebral performance category; ROSC, return of spontaneous circulation; SMD, standardised mean difference.

### Discharge diagnoses among hospitalised cases of OHCA

**Figure 2:**
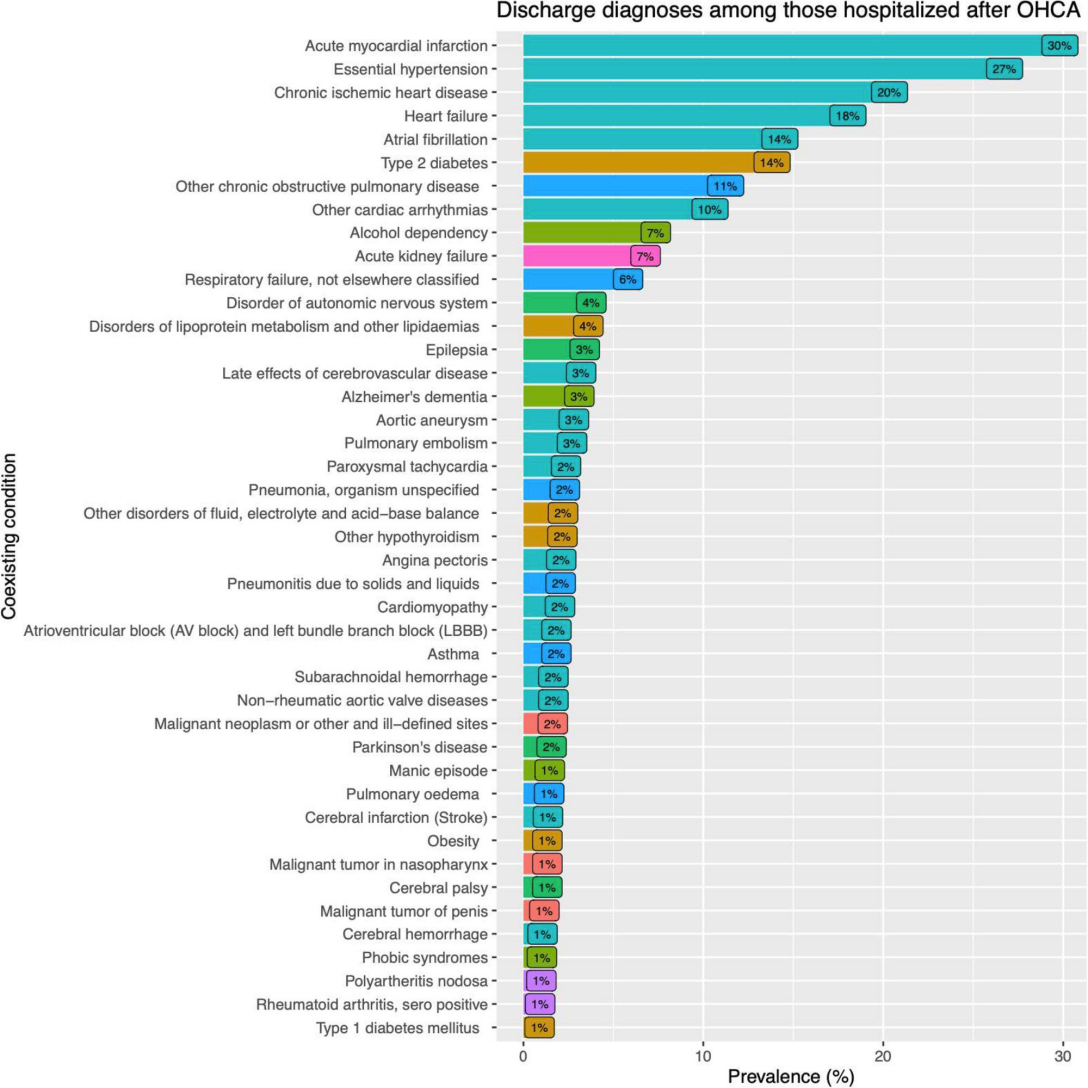
Discharge diagnosis among patients hospitalised for out-of-hospital cardiac arrest (OHCA).

Figure 2 presents acute diagnoses established at hospital discharge, irrespective of survival. Among these initial survivors, 30% were diagnosed with acute myocardial, 27% with hypertension, 20% with chronic ischaemic heart disease, 18% with heart failure and 14% with atrial fibrillation. The top five discharge diagnoses were cardiovascular conditions. Type 2 diabetes was prevalent in 14% of cases, and COPD in 11%. Other cardiac arrhythmias (besides atrial fibrillation and flutter) were prevalent in 10% of initial survivors. Beyond these diagnoses, only alcohol dependency and acute kidney injury were more frequent than 5% (figure 2).

Other notable discharge diagnoses were pulmonary embolism (3%), aortic aneurysm (3%), paroxysmal tachycardias (2%), cardiomyopathies (2%), subarachnoidal haemorrhage (2%) and type 1 diabetes (1%) (figure 2).

### Survival OHCA and heart failure diagnosis at discharge

**Figure 3:**
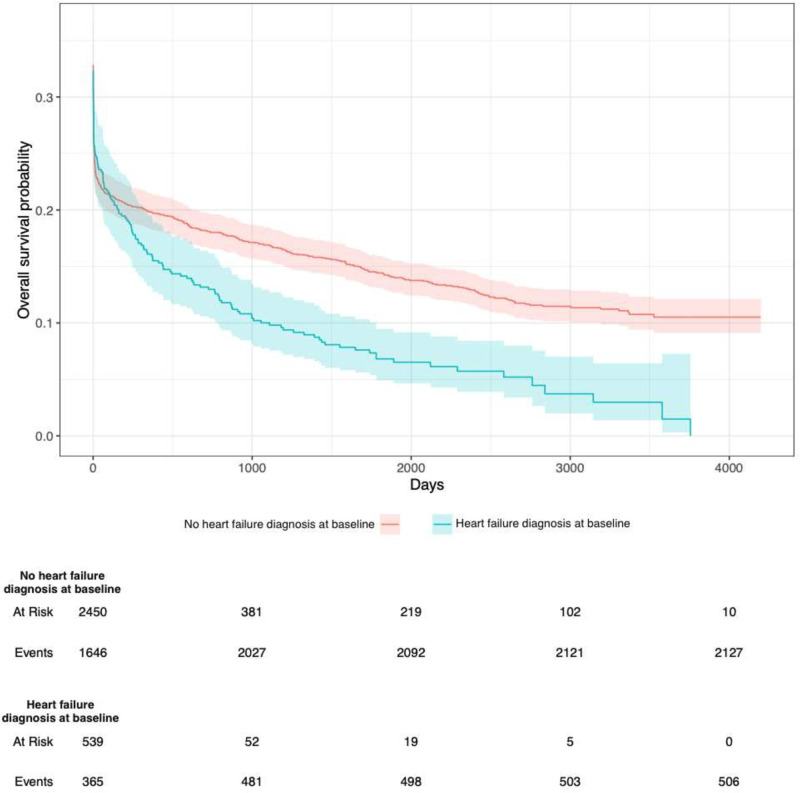
Kaplan-Meier plots illustrating hospitalised patients’ survival in relation to baseline heart failure history. Blue=heart failure diagnosis at baseline. Red=no heart failure diagnosis at baseline. Survival probability in regard to death is shown on the y-axis. Time in days is shown on the x-axis.

Figure 3 presents overall survival probability in relation to heart failure diagnosis. Among those who were hospitalised, the vast majority died early (within 24 hours). For patients with a heart failure diagnosis at discharge, early survival was somewhat higher than those without heart failure diagnosis at discharge. However, survival curves, crossed after approximately 100 days, such that those with a discharge diagnosis of heart failure had clearly worse long-term prognosis. After roughly 1000 days, survival curves were proportional for those with and without heart failure at discharge (figure 3).

### Survival OHCA and AMI diagnosis at discharge

**Figure 4:**
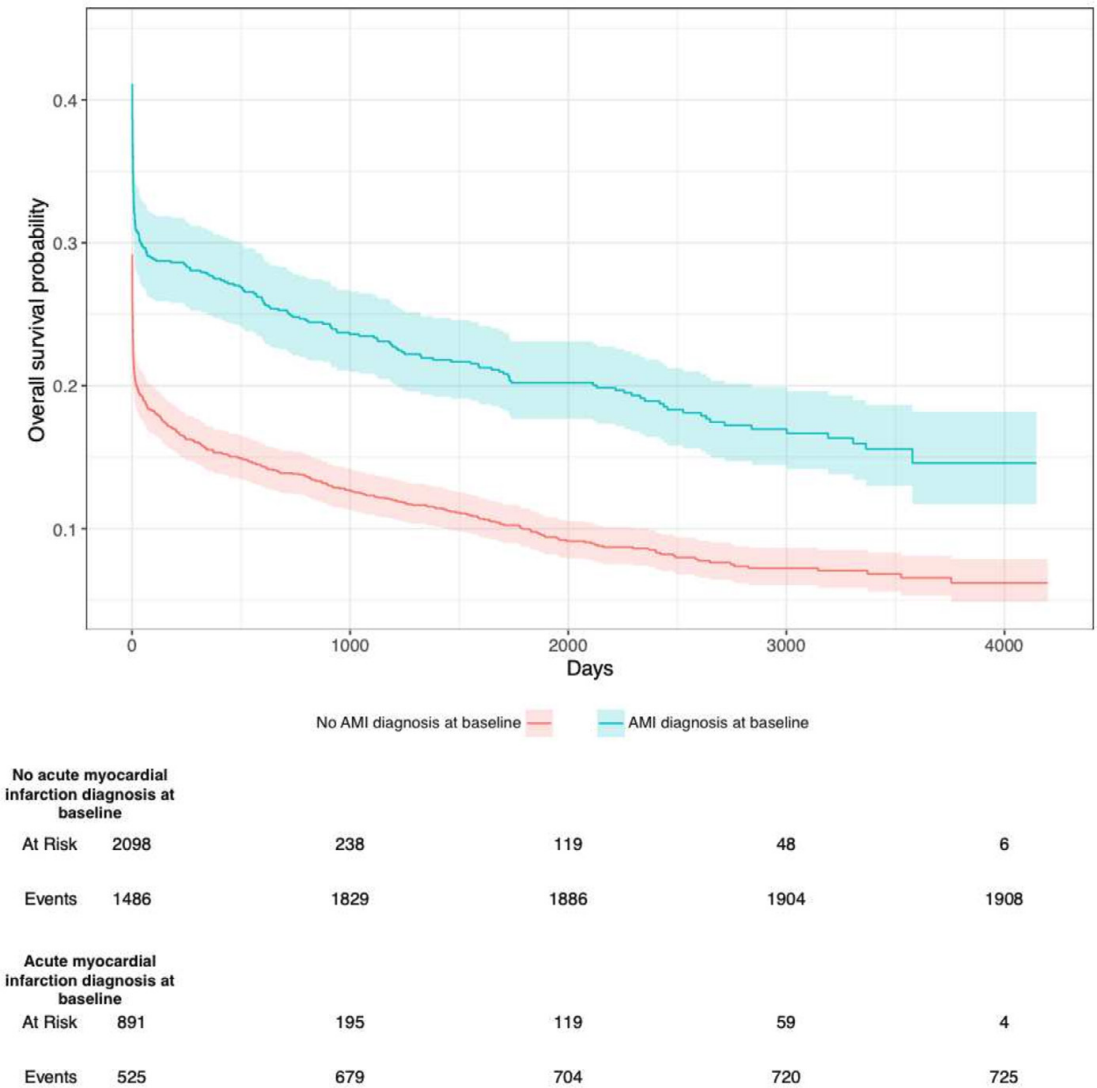
Kaplan-Meier plots illustrating hospitalised patients’ survival in relation to baseline acute myocardial infarction (AMI) history. Blue=AMI diagnosis at baseline. Red=no AMI diagnosis at baseline. Survival probability in regard to death is shown on the y-axis. Time in days is shown on the x-axis.

Figure 4 presents overall survival probability in relation to AMI diagnosis at discharge.

## Discussion

It is previously stated that approximately 30%–50% of all sudden cardiac deaths results from coronary artery disease.^13 14^ This nationwide study shows that roughly 30% of all cases of OHCA that are admitted to hospital are diagnosed with AMI. While AMI was the most common condition diagnosed, 30% is far from a majority of cases. Chronic ischaemic heart disease was diagnosed in 20% of all cases, the majority overlapping with those receiving a diagnosis of AMI, making ischaemic heart disease relevant in majority of cases of OHCA today. Furthermore, our results demonstrated that the most common previous conditions prior to cardiac arrest were cardiovascular comorbidities such as hypertension, heart failure, ischaemic heart disease and atrial fibrillation. While heart failure affects around 1%–2% of the adult population, we observe more than 10-fold higher rates in the OHCA studied population.^17–19^

Perhaps the most striking finding of the current study is evident in figure 2, which shows that all top seven discharge diagnosis represent lifestyle conditions that can be prevented using cheap and readily available drugs (statins, antihypertensives, aspirin, etc). Moreover, given the recent breakthroughs in obesity treatment with GLP-1 receptor antagonists, these conditions (obesity and its downstream effectors) are becoming easier and more feasible to treat. It follows that a broader and more aggressive risk factor management may be crucial to prevent at-risk individuals from developing cardiac arrests.

The study is based on high-quality registries with nationwide coverage. Yet, there remains a risk for selection bias due to several facts. First, the SRCR only includes cases of OHCA where resuscitation was attempted. This excludes many patients with sudden cardiac arrest who the EMS deemed could not be resuscitated. Nevertheless, the SRCR has always aimed to only study cases in whom resuscitation is attempted and deemed to be potentially life-saving. The implications of this is discussed in Hirlekar *et al*, which showed that patients who receive CPR have lower comorbidity than those who did not.^20^ Moreover, our final diagnoses are actually discharge diagnoses established among cases who were admitted to the hospital. This hampers our ability to extrapolate our discharge diagnoses to the entire cohort. With regard to our main finding, however, cases with AMI are known to have higher survival than the average OHCA population. This could be explained by higher rates of shockable rhythm and relatively lower age. Hence, the prevalence of coronary artery disease should be higher among survivors compared with the whole OHCA population. Yet, we find unexpectedly low rates of AMI and ischaemic heart disease among survivors.

These results are also in line with a recent study from the SRCR, which showed that in the period 2017–2020, compared with the early 1990s, the probability of OHCA due to cardiac causes, as well as the probability of exhibiting a shockable rhythm, was halved. This dramatic reduction is believed to be caused by reductions in the rate of cardiovascular disease in Western countries.^21^

The majority of patients die immediately after OHCA. Patients diagnosed with AMI at discharge display lower mortality throughout, without any change in hazard over time. However, patients discharged with heart failure diagnosis exhibit higher survival initially (presumably due to higher likelihood of shockable rhythm), but then a non-proportional hazard over time, such that their mortality surpassed the other cases after roughly 100 days. This is likely an impact of ventricular function, which is known to deteriorate rapidly in patients with heart failure. With regard to cases with an AMI, the better prognosis could be explained by successful PCI, which can reverse the underlying substrate for the cardiac arrest.

In summary, we find evidence that nowadays a minority of cardiac arrests are due to coronary artery disease and myocardial infarction and its complications. All top seven discharge diagnoses among survivors suggest that a substantial proportion of cardiac arrests could be prevented by treating traditional risk factors for cardiovascular disease.

## Data Availability

Data are available upon reasonable request. Data sharing is allowed after approval from the Swedish Ethical Review Authority. Please contact the Swedish Cardiopulmonary Resuscitation Registry for more information.
