# Supplementary material for: Comorbidities prior to out-of-hospital cardiac arrest and diagnoses at discharge among survivors
Source: Open Heart. 2023 Nov 14;10(2):e002308. doi: 10.1136/openhrt-2023-002308 (PMC10649799; doi:10.1136/openhrt-2023-002308)
Supplement: Supplementary data [file openhrt-2023-002308supp001.pdf]

Table 1. Baseline characteristics in 54484 patients with out of hospital cardiac arrest

var	Men	Women	p		SMD
n	35894	18590			
**SOCIOECONOMIC STATUS**					
Disposable income of family [median IQR]	2671.00 [1670.00, 4043.75]	1906.50 [1392.00, 3195.00]	<0.001		0.167
Disposable income of patient [median IQR]	1680.00 [1336.00, 2417.00]	1393.00 [1121.00, 1695.00]	<0.001		0.181
**WORK OR PROFESSION**					0.475
Senior officials and senior positions	1003 (3.1)	158 (0.9)			
Qualified officials	1374 (4.3)	448 (2.7)			
Other officials	584 (1.8)	400 (2.4)			
Small business	1536 (4.8)	265 (1.6)			
Supervisors and technicians	43 (0.1)	0 (0.0)			
Vocationally trained in trade service and care	736 (2.3)	844 (5.0)			
Vocationally trained workers	1297 (4.0)	42 (0.2)			
Other workers	2094 (6.5)	380 (2.3)			
Not employed	23510 (73.1)	14271 (84.9)			
Unknown	0 (0.0)	0 (0.0)			
**Educational level**			<0.001		0.297
Pre gymnasium 9 years	8738 (26.7)	5093 (30.0)			
Pre gymnasium 9 years	4075 (12.5)	2189 (12.9)			
Gymnasium 3 years	8480 (25.9)	5395 (31.8)			
Gymnasium 3 years	4948 (15.1)	1213 (7.1)			
Post gymnasium 3 years	2638 (8.1)	1188 (7.0)			
Post gymnasium 3 years or longer	2864 (8.8)	1344 (7.9)			
Research education	281 (0.9)	43 (0.3)			
Unknown	657 (2.0)	521 (3.1)			
**Marital status**			<0.001		0.569
Not married	8263 (24.8)	3060 (17.7)			
Married	16935 (50.9)	6134 (35.5)			
Surviving partner	2 (0.0)	1 (0.0)			
Registered partner	15 (0.0)	1 (0.0)			
Divorced	5291 (15.9)	3176 (18.4)			
Divorced partner	9 (0.0)	2 (0.0)			
Widow widower	2782 (8.4)	4890 (28.3)			
**PREVIOUS CONDITIONS**					
Arthrosis any	5056 (14.1)	3548 (19.1)	<0.001		0.135

Table 1. Baseline characteristics in 54484 patients with out of hospital cardiac arrest

var	Men	Women	p	SMD
Epilepsy	5552 (15.5)	2644 (14.2)	<0.001	0.035
Tumour of unknown origin in GI tract	4960 (13.8)	2855 (15.4)	<0.001	0.044
Alcohol dependency	5391 (15.0)	2357 (12.7)	<0.001	0.068
Pneumonia any	4605 (12.8)	2627 (14.1)	<0.001	0.038
Pharyngeal tumour benign	3781 (10.5)	2938 (15.8)	<0.001	0.156
Phobic disorders	3329 (9.3)	2572 (13.8)	<0.001	0.143
Affective disorders	3101 (8.6)	2600 (14.0)	<0.001	0.169
Pneumonia bacterial	2546 (7.1)	1548 (8.3)	<0.001	0.046
Alzheimers dementia	2199 (6.1)	1885 (10.1)	<0.001	0.147
Bechterew disease	2216 (6.2)	1679 (9.0)	<0.001	0.108
Malignant melanoma	2538 (7.1)	1337 (7.2)	0.614	0.005
Other disorders of fluid, electrolyte and acid-base balance	1867 (5.2)	1814 (9.8)	<0.001	0.174
**SPATIOTEMPORAL INFORMATION**				
**Time of cardiac arrest**			<0.001	0.067
0 to 6 am	4831 (16.1)	2626 (17.0)		
1 to 6 pm	9381 (31.3)	4365 (28.3)		
7 to 11 pm	5657 (18.9)	3031 (19.6)		
7 to 12 am	10092 (33.7)	5421 (35.1)		
**Location of cardiac arrest**			<0.001	0.315
Home	24253 (67.8)	14580 (78.8)		
Public place	7127 (19.9)	1691 (9.1)		
Other places	4368 (12.2)	2235 (12.1)		
**Location of arrest in public place**			<0.001	0.292
Shopping centre	380 (6.8)	115 (8.8)		
Train station	132 (2.3)	33 (2.5)		
Park	917 (16.3)	179 (13.8)		
Work place	415 (7.4)	83 (6.4)		
Swimming facility or beach	202 (3.6)	71 (5.5)		
Airport	43 (0.8)	9 (0.7)		
Street	2652 (47.2)	670 (51.5)		
Church mosque synagogue	54 (1.0)	26 (2.0)		
Amusement park	130 (2.3)	36 (2.8)		
Sports facility	625 (11.1)	61 (4.7)		
Other	71 (1.3)	17 (1.3)		
**Location of arrest in other places**			<0.001	0.494
Ambulance	1466 (34.5)	924 (42.5)		

Table 1. Baseline characteristics in 54484 patients with out of hospital cardiac arrest

var	Men	Women	p	SMD
Hotel room	61 (1.4)	20 (0.9)		
Private office	84 (2.0)	7 (0.3)		
Care facility	757 (17.8)	672 (30.9)		
Other	1677 (39.5)	453 (20.8)		
Primary care jourcentral	108 (2.5)	45 (2.1)		
Dental clinic	52 (1.2)	35 (1.6)		
Hospital without rescue team	40 (0.9)	19 (0.9)		
**PREHOSPITAL INTERVENTIONS**				
Bystander CPR	19215 (55.5)	9620 (53.9)	0.001	0.032
Bystander CPR old variable	9062 (58.8)	4186 (54.2)	<0.001	0.092
AED connected by bystander	1330 (7.4)	489 (5.1)	<0.001	0.093
AED used by bystander	527 (41.0)	113 (23.7)	<0.001	0.377
Compressions used	34047 (95.2)	17663 (95.3)	0.596	0.005
Mechanical compressions	14325 (41.6)	6741 (37.9)	<0.001	0.076
Ventilation provided	33023 (92.6)	17080 (92.5)	0.696	0.004
Intubation performed	10217 (29.0)	4936 (27.0)	<0.001	0.044
Laryngeal mask placed	13011 (62.2)	6518 (58.9)	<0.001	0.069
Defibrillated	13276 (38.3)	4196 (23.6)	<0.001	0.323
Defibrillated number - mean (SD)	3.68 (3.26)	2.82 (2.73)	<0.001	0.286
Epinephrine administered	28103 (79.2)	14345 (78.2)	0.006	0.025
Amiodarone administered	5081 (14.5)	1143 (6.3)	<0.001	0.271
**CRITICAL TIME INTERVALS [median, IQR]**				
Time from arrest to EMS dispatch	2.00 [1.00, 5.00]	2.00 [1.00, 6.00]	<0.001	0.043
Time from arrest to CPR start	3.00 [0.00, 10.00]	2.00 [0.00, 10.00]	<0.001	0.033
Time from arrest to defibrillation	14.00 [8.00, 23.00]	16.00 [9.00, 26.00]	<0.001	0.119
Time from arrest to EMS arrival	13.00 [8.00, 20.00]	13.00 [8.00, 20.00]	<0.001	0.044
Time from EMS dispatch to arrival	10.00 [7.00, 16.00]	10.00 [7.00, 16.00]	0.933	0.002
Time from arrest to ROSC	15.00 [9.00, 23.00]	15.00 [8.00, 23.00]	0.016	0.026
**INITIAL PRESENTATION**				
Consciousness on EMS arrival at scene	3564 (10.2)	2064 (11.4)	<0.001	0.040
**Breathing on EMS arrival at scene**			0.007	0.032
No breathing	27276 (78.1)	13857 (76.9)		
Agonal breathing	3704 (10.6)	1981 (11.0)		

Table 1. Baseline characteristics in 54484 patients with out of hospital cardiac arrest

var	Men	Women	p	SMD
Normal breathing	3918 (11.2)	2182 (12.1)		
Unknown	15 (0.0)	7 (0.0)		
Pulse on EMS arrival at scene	4596 (13.5)	2657 (15.1)	<0.001	0.046
Spontaneous circulation on hospital arrival	9161 (43.3)	4759 (48.3)	<0.001	0.100
Consciousness on hospital arrival	2461 (11.9)	878 (9.1)	<0.001	0.091
**CIRCUMSTANCES AT TIME OF ARREST**				
Witnessed cardiac arrest any Witnessed	22782 (65.4)	11544 (63.9)	0.001	0.030
Witnessed by ambulance	2367 (19.9)	1483 (24.2)	<0.001	0.105
Telephone CPR	6055 (62.0)	3266 (63.8)	0.038	0.036
**Bystander education**			0.061	0.047
Laymen not CPR educated	3436 (47.1)	1809 (46.0)		
Laymen CPR educated	2638 (36.2)	1509 (38.3)		
Health care professional	1219 (16.7)	618 (15.7)		
Ambulance first on scene	14069 (73.6)	7694 (76.2)	<0.001	0.061
Emergency service first on scene	5888 (32.0)	2851 (29.4)	<0.001	0.055
Police first on scene	915 (5.0)	415 (4.3)	0.010	0.033

Table 1. Baseline characteristics in 54484 patients with out of hospital cardiac arrest

var	Men	Women	p	SMD

ICD-codes

C43	Malignant melanoma
C60	Malignant neoplasm of male genital organs
C76	Malignant neoplasm of other and ill-defined sites
D10	Pharyngeal tumour benign
D37	Tumor of unknown origin in GI tract
E03	Other hypothyroidism
E10	Type 1 diabetes mellitus
E11	Type 2 diabetes mellitus
E14	Unspecified diabetes mellitus
E66	Overweight and Obesity
E78	Disorders of lipoprotein metabolism an other lipaemias
E86	Volume depletion
E87	Other disorders of fluid, electrolyte and acid-base balance
F00	Alzheimers dementia
F10	Alcohol dependency
F30	Manic episode
F40	Phobic disorders
G20	Parkinsons disease
G40	Epilepsy
G50	Trigeminal neuralgia
G80	Cerebral palsy
G90	Disorder of the autonomic nervous system
I10	Essential hypertension
I20	Angina pectoris, including unstable angina
I21	Acute myocardial infarction
I25	Chronic ischemic heart disease
I26	Pulmonary embolism
I35	Non-rheumatic aortic valve disorders
I42	Cardiomyopathy
I44	Atrioventricular and left bundle-branch block
I46	Cardiac arrest
I47	Paroxysmal tachycardia
I48	Atrial fibrillation
I49	Other cardiac arrhytmias
I50	Heart failure
I60	Subarachnoidal haemorrage
I61	Intracerebral hemorrhage
I63	Cerebral infarction (Stroke)
I69	Sequele of cerebrovascular disease
I70	Atherosclerotic disease

I71	Aortic aneurysm and dissection
I73	Other peripheral vascular diseases
I80	Phlebitis and trombophlebitis
I95	Hypotension
J06	Acute upper respiratory infections of multiple and unspecified sites
J15	Bacterial pneumonia, not elsewhere classified
J18	Pneumonia, unspecified organism
J20	Acute bronchitis
J44	Other chronic obstructive pulmonary disease
J45	Asthma
J69	Pneumonitis due to food and vomit
J81	Pulmonary oedema
J96	Respiratory insufficiency, not elsewhere classified
K50	Crohns disease
M15	Arthrosis, any
M30	Polyarteritis nodosa and related conditions
M45	Bechterew disease
N17	Acute kidney failure

ATC-codes

ATC A10 = Antidiabetic drugs
ATC B01 = Anticoagulant or antiplatelet agent
ATC C03 = Diuretics
ATC C07 = Beta blockers
ATC C08 = Calcium channel blockers
ATC C09 = ACE inhibitor or ARB
ATC C10 = Lipid lowering drugs

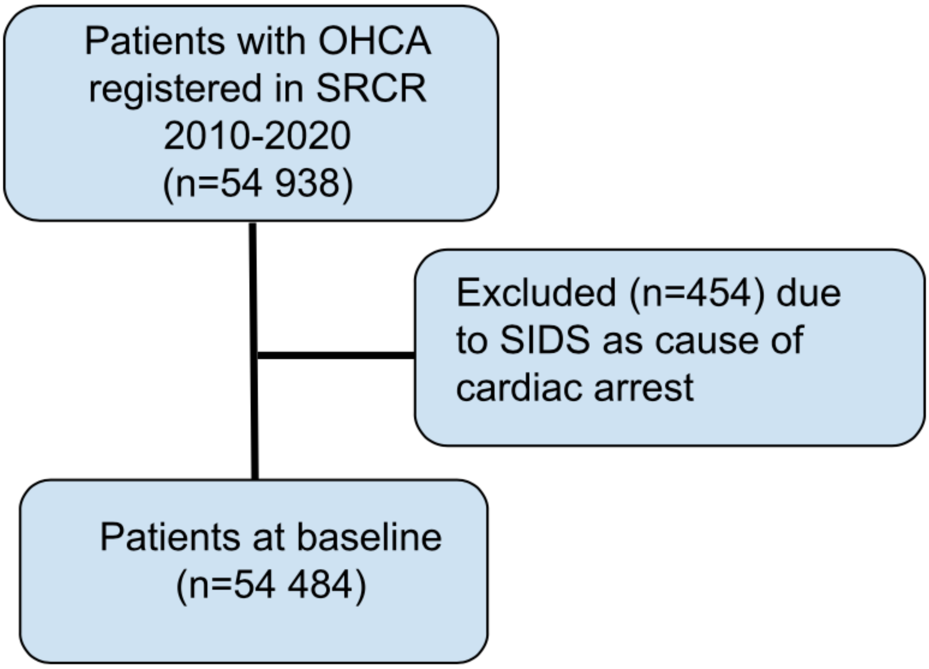
Patients with OHCA registered in SRCR 2010-2020 (n=54 938)
Excluded (n=454) due to SIDS as cause of cardiac arrest
Patients at baseline (n=54 484)

Flowchart
